# Cadmium Transport in a Model of Neonatal Intestinal Cells Correlates to MRP1 and Not DMT1 or FPN1

**DOI:** 10.1155/2013/892364

**Published:** 2013-01-27

**Authors:** Helena Öhrvik, Eva Tydén, Per Artursson, Agneta Oskarsson, Jonas Tallkvist

**Affiliations:** ^1^Department of Biomedical Sciences and Veterinary Public Health, Swedish University of Agricultural Sciences, Box 7028, 75007 Uppsala, Sweden; ^2^Department of Pharmacy, Uppsala University, Box 580, 75123 Uppsala, Sweden

## Abstract

Newborns have a higher gastrointestinal uptake of cadmium than adults. In adults, the iron transporters DMT1 and FPN1 are involved in the intestinal absorption of cadmium, while in neonates, the mechanisms for cadmium absorption are unknown. We have investigated possible cadmium transporters in the neonatal intestine by applying a model of immature human intestinal epithelial Caco-2 cells. To mimic the continuous cadmium exposure via diet in neonates, cells were allowed to differentiate for 7 days in medium containing 1 **μ**M CdCl_2_. A dramatic upregulation of the MT1 gene expression followed cadmium pretreatment, indicating a high sensitivity of the immature cells to cadmium. Cadmium pretreatment increased the basolateral efflux of ^109^Cd, without causing any effects on the passive diffusion of mannitol or the transepithelial electrical resistance. The augmented transport of cadmium was correlated to an upregulation of MRP1 gene expression and increased activity of the efflux protein MRP1. No effects were observed on gene expression of the efflux proteins MRP2 and P-gp or the iron transporters DMT1, DMT1-IRE and FPN1. In conclusion, our data indicate that continuous cadmium exposure increases the absorption of the metal in immature intestinal cells and that MRP1 is involved in the intestinal cadmium absorption in newborns.

## 1. Introduction

Cadmium is a ubiquitous toxic metal that has no known biological functions but causes several adverse health effects in humans. Neonates are more sensitive to cadmium toxicity than adults, due to a higher gastrointestinal absorption [1–3] and the potential of the element to interfere in the development of the central nervous system [4]. The main source for cadmium exposure in infants is cereal and soy-based infant formulas and weaning foods [3].

The intestinal absorption of cadmium in adults is generally low but increases during iron deficiency [5–9], suggesting that dietary cadmium is absorbed in the intestine by the same mechanism as non-heme iron. The major transporters responsible for the absorption of nonheme iron are the apical influx divalent metal transporter 1 (DMT1) and the basolateral efflux ferroportin 1 (FPN1) [10–12]. DMT1, the isoform DMT1-IRE (containing an iron responsive element) and FPN1 expressions are induced in human adults during iron deficiency and repressed under iron sufficient conditions [13, 14]. In addition, studies in adult rats have demonstrated a strong correlation between cadmium absorption and duodenal expressions of DMT1 and FPN1 [15, 16]. However, in newborns the possible role of DMT1 and FPN1 in cadmium absorption has not been clarified. It has been demonstrated that the expression and localization of DMT1 and FPN1 in duodenal enterocytes is not affected by iron status, neither in suckling piglets at postnatal day 16 nor in suckling rat pups at postnatal day 10, indicating an immature regulation of iron [17, 18]. Thus, cadmium absorption seems to be regulated by other mechanisms at this developmental stage. 

Gastrointestinal cadmium absorption may also be mediated by proteins belonging to the ATP binding cassette (ABC) superfamily of transporters [19]. Multidrug resistance associated protein 1 (MRP1) is a transporter involved in the basolateral efflux of compounds out of enterocytes into the systemic circulation [20]. Cadmium upregulates MRP1 and the protein have been demonstrated to mediate transport of the metal in functional yeast experiments [21, 22]. The efflux proteins MRP2 and P-glycoprotein (P-gp) located at the apical membrane of enterocytes are also upregulated by cadmium [23, 24] and may thus influence the bioavailability of the metal. 

Repeated cadmium treatment has previously been shown to increase cadmium absorption compared to a single treatment both *in vivo* and in intestinal *in vitro* preparations of adult rats [25]. However, even though MT involvement has been suggested, the cellular mechanism for how cadmium enhances its own absorption remains to be elucidated [25]. Intestinal MT is induced by cadmium [26], but there are numerous reports concluding that the impact of MT on the intestinal cadmium absorption is of minor importance [27]. It appears that the predominant role of this cytosolic small molecular weight metal binding protein is to protect the cells from cytotoxic effects [28].

Twenty-one-day postconfluent Caco-2 cells, which are fully differentiated (mature), have been extensively used as an *in vitro* model of the adult human intestinal epithelium [29]. Under normal cell culture conditions, Caco-2 cells spontaneously differentiate into a phenotype that resembles polarized absorptive enterocytes [30–32]. The Caco-2 model has been widely used to investigate cadmium absorption and effects [33–38]. Mature Caco-2 cells treated with iron have reduced uptake and transport of cadmium that correlates to iron-induced decrease in DMT1 levels [37]. In concordance, a reduced cadmium uptake was demonstrated in Caco-2 cells subjected to molecular DMT1 knock down [33]. The ABC efflux transporters MRP1, MRP2, and P-gp are present in Caco-2 cells [39, 40] and the expression of P-gp in these cells has been shown to be induced by long-term cadmium treatment [23]. 

The present study was undertaken to investigate possible transporters involved in the absorption of cadmium in the neonatal intestine and the impact of cadmium pretreatment. To mimic the continuous cadmium exposure in neonates via milk and/or formula, confluent Caco-2 cells were allowed to differentiate during 7 days (referred to as “immature Caco-2 cells”) in the presence and absence of cadmium. Apical to basolateral transport, studies with cadmium were performed and gene expressions of the transporters MRP1, MRP2, P-gp, DMT1, DMT1-IRE, FPN1, and of MT were quantified as well as the MRP1 activity. 

## 2. Materials and Methods

### 2.1. Chemicals

Cadmium chloride and nitrilotriacetic acid were purchased from Sigma Aldrich. Dulbeccos' modified Eagle medium (DMEM), heat inactivated fetal calf serum, nonessential amino acids for modified Eagle medium (MEM), N-2-hydroxyethyl-piperazine-N′-2-ethanesulfonic acid (HEPES) buffer 1 M, nonessential amino acids (100x), and gentamycin were purchased from Invitrogen via VWR International, LLC. Apo-transferrin (tissue culture grade) was obtained from Calbiochem via VWR International, LLC. [^14^C]-mannitol (specific activity 2.09 GBq/mmol) was purchased from PerkinElmer and ^109^Cd (specific activity 15.2 GBq/mg Cd^2+^) from GE Healthcare. Ultima Gold (scintillation liquid) was obtained from Chemical Instruments AB. Calcein acetoxymethyl ester (calcein AM) fluorescent dye was purchased from BD Biosciences. Hank's balanced salt solution (HBSS) and all other chemicals used, but not specified in the experiments, were of analytical grade and from VWR International, LLC. 

### 2.2. Cell Culture and Treatment

Human intestinal Caco-2 cells were obtained from American Cell Culture Collection (Rockville, USA). The cells were cultured as previously described [41] with some minor modifications. Briefly, the Caco-2 cells were expanded in normal tissue culture flasks in DMEM supplemented with 10% fetal calf serum (Invitrogen) and 1% gentamycin (50 mg/mL, Invitrogen). The cells were seeded onto permeable polycarbonate filters at a density of 2.5 × 10^5^ cells/filter support (Ø 12 mm, pore radius 0.4 *μ*m, Transwell, Corning), and apical medium was changed 8 h after seeding to remove excess of cells and to reduce the risk of multilayer formation [42]. The Caco-2 cells were then allowed to differentiate for 7 days postconfluency. The medium was changed every second day, and the cells were examined daily by reverse phase microscopy for confluence, shape of cells, and dome formations. Cells of passage numbers 97–100 were used in the experiments. 

Each cell monolayer was randomly assigned to differentiate for 7 days postconfluency in control (C) medium or medium supplemented with 1 *μ*M cadmium (CdCl_2_ × 1H_2_O) (Cd). Previous investigations have demonstrated that the viability of the Caco-2 cells at various stages of differentiation is not affected by 1 *μ*M cadmium treatment [26]. 

### 2.3. Assessment of Monolayer Integrity

The integrity of the control and cadmium pretreated monolayers was examined by determining the permeability of the hydrophilic marker ^14^C-mannitol, mainly as described [42]. Briefly, cell monolayers were rinsed with prewarmed Hank's Balanced Salt Solution pH 7.4 (HBSS) and then incubated in HBSS at 37°C for 20 min. The HBSS was discarded from the apical chambers prior to transferring the filters to a fresh and prewarmed plate containing 1.5 mL 37°C HBSS in the basolateral chambers. HBSS (0.5 mL, pH 6.3) prewarmed to 37°C and containing 0.14 *μ*Ci/mL ^14^C-mannitol was added to the apical chambers. The transport experiment was carried out at 37°C under gentle shaking (50 rpm) for 3 h. Every 30 min the filters with the monolayers were transferred to new wells containing 37°C HBSS and samples from both the apical and basolateral chambers were taken for subsequent determination of radioactivity by *β*-spectrometry (Tricarb 1900 CA, Packard). The integrity of the monolayers was also verified by measuring the net transepithelial electrical resistance (TEER) across the monolayers, as described [42]. Resistance across monolayers was measured in control and cadmium supplemented media (pH 7.4, 37°C), and background resistance was measured across filters without cells.

### 2.4. ^109^Cd Transport and Uptake

Cell monolayers, differentiated for 7 days postconfluency in control medium or medium supplemented with cadmium were used for uptake (from apical chamber into the cells) and transport (from apical chamber via the cells into the basolateral chamber) experiments. The experiments were performed at 37°C, pH 7.4, in sterile filtered HBSS, buffered with 25 mM HEPES, mainly as previously described [41]. Briefly, the monolayers were first rinsed in prewarmed HBSS and then incubated in HBSS at 37°C for 20 min before being transferred to new wells where fresh, 37°C HBSS was added to the apical (0.5 mL, pH 6.3) and basolateral (1.5 mL, pH 7.4, supplemented with 15 *μ*M human apo-transferrin) chambers. The apical solution was supplemented with 30 nM ^109^Cd (1 *μ*Ci/mL). To determine the transport of ^109^Cd across the monolayers 1.4 mL, samples were taken from the basolateral chambers at 30 min intervals for 3 h and at the same time the cell monolayers were transferred to new wells containing prewarmed HBSS as described above. The radioactivity in the collected samples was measured by *γ*-spectrometry (Cobra, Auto-gamma, Packard). To determine the cellular uptake of ^109^Cd from the apical chamber following the 3 h incubation period, the filters with the monolayers were rinsed in 4°C HBSS for 5 min and then dissolved in 1 mL 0.5 M NaOH for 48 h, followed by *γ*-spectrometry of an aliquote of the lysate. In order to reduce the unstirred water layer during the transport and uptake experiments, a plate shaker was used to agitate the monolayers at a rate of 50 rpm. The experiments were reproduced two times. 

### 2.5. Quantitative Gene Expression

Total RNA from Caco-2 cells treated as described above prior to the transport experiments was isolated by the use of NucleoSpin RNA II kit containing Dnase I, according to the instructions of the manufacturer (Macherey-Nagel, Germany). The cells were rinsed with ice cold PBS prior to the addition of lysis buffer and the isolation of RNA. The integrity of the RNA was confirmed by gel electrophoresis, and quantification of the RNA was performed according to the RNA-specific Quant-iT RiboGreen protocol (Invitrogen). RNA was stored at −70°C until used for quantitative gene expression analyses.

Quantification of gene expressions of the proteins presented in Table 1 was performed by one step real-time RT-PCR by applying a Rotorgene, RG 3000 instrument (Corbett Research, Australia) and QuantiTect SYBR Green RT-PCR (Qiagen) reagents, mainly according to the manufacturer's instructions. Gene expression was normalized to total RNA. Primer concentration was 0.4 *μ*M, and 200 ng total RNA was used as template. Gene-specific primers for the human genes were designed by using NCBI primer design, and all gene products were sequenced and compared against the human genome by applying the UCSC Human Genome Browser (Table 1). Triplicate 25 *μ*L volume RT-PCR reactions were used under the following PCR cycling conditions. Step 1: 50°C 30 min; Step 2: 95°C 15 min; Step 3: 94°C 60 sec; Step 4: 55°C 60 sec; Step 5: 68°C 45 s; Step 6: 35 or 40 repeats of steps 3–5; Step 7: 68°C 7 min. In the analyses, five known concentrations of the human genes and a nontemplate control served as internal controls. Melt curve analysis was performed for each sample to check the specificity of the obtained PCR products. Relative quantification of mRNA expressions was performed as previously described [43]. Quantification of gene expressions as well as analyses of melt curves were carried out by the use of Rotorgene Software (Corbett Research, Australia).

### 2.6. Functional Activity: MRP-Mediated Efflux of Calcein

To measure the functional activity of MRP transporter proteins following cadmium pretreatment, an efflux assay with calcein was performed. Calcein AM is a lipophilic, nonfluorescent dye that rapidly enters cells through the plasma membrane. Once inside the cell, endogenous esterases cleave the AM ester bond releasing calcein, a hydrophilic, highly fluorescent dye that becomes trapped inside the cell. The ABC transporters MRP1 and MRP2 have been shown to transport both calcein AM and calcein. In contrast, P-gp transporter proteins efflux the nonfluorescent calcein AM, but not the fluorescent calcein from the cells into the apical chamber [44]. MRP1 transport proteins efflux both the nonfluorescent calcein AM and the fluorescent calcein from the cells across the basolateral membrane, and MRP2 transports both calcein AM and calcein although across the apical membrane into the apical chamber [45].

Cells were seeded onto filters and allowed to differentiate in medium supplemented with Cd as described above. The monolayers were washed as described above in 37°C HBSS, buffered with 25 mM HEPES before loading the cells with 1.3 *μ*M calcein AM solved in 1% DMSO and added both to the apical and basolateral chambers. The loading of the cells with the permeable calcein AM was conducted at 7°C, to reduce the active efflux by P-gp and MRPs of the compound, for 1 h in HBSS. Two quick washes in 7°C HBSS were performed after loading to remove excessive calcein AM and then the monolayers were transferred to new wells with 37°C HBSS, starting the efflux study. During the 2 h efflux study, samples were withdrawn every 30 min from the basolateral chambers. At the end of the experiment, samples were withdrawn also from the apical chambers. The samples were immediately transferred to black 96 plate wells and fluorescence was measured instantly at 490 nm (excitation) and at 520 nm (emission) by a multiplate reader. During the loading and efflux experiment, a plate shaker was used to gently agitate the monolayers at a rate of 50 rpm as described above. The functional assay of MRP-mediated calcein efflux was reproduced two times. 

### 2.7. Statistical Analyses

The data are presented as mean ± SEM, unless otherwise stated. The apparent permeability coefficient for mannitol was calculated as *P*_app_ = (d*Q*/d*t*)(1/(*AC *_0_)), where d*Q*/d*t* is the steady state flux (dpm/s), *A* is the filter area (cm^2^), and *C *_0_ is the initial concentration in the apical chamber (*μ*M). The statistical software Minitab release 15 (Minitab Inc.) was used for calculations of basic descriptive statistics and analysis of variances (ANOVA). Data were tested for homogeneity of variances by Bartlett's test. Data on cadmium transport were log transformed before statistical analysis. ANOVA was used to investigate the effects of cadmium on the dependent variables (cadmium uptake and transport, MRP activity, DMT1, DMT1-IRE, FPN1, MRP1, MRP2, P-gp, and MT1 gene expressions), followed by Tukey's test to determine differences between means. The level of significance was set at *P* ≤ 0.05, two-sided. 

## 3. Results

### 3.1. Assessment of Monolayer Integrity and Histology of the Immature Caco-2 Cells

The passive diffusion of ^14^C-mannitol across the monolayers was linear during the 3 h experimental period. The apparent permeability coefficient (*P*_app_) for mannitol across the control monolayers was 1.15 ± 0.3 × 10^−6^ cm/s (mean ± SD; *n* = 3), demonstrating that the 7 days postconfluent cells had developed a barrier. Cadmium pretreatment did not influence the apparent permeability for mannitol (*P* = 0.60). The TEER across the control monolayers was 275 ± 18 Ω∗cm^2^ (mean ± SEM; *n* = 6). Background resistance across filters in control and cadmium supplemented media (pH 7.4, 37°C) without cells was about 80 Ω∗cm^2^. Cadmium pretreatment did not affect the TEER value for the monolayers (*P* = 0.70).

The histological examination of the sectioned immature Caco-2 monolayers did not reveal any difference between controls and cadmium pretreated cells. Dome formations were observed in all monolayers at the end of the 7 days differentiation period. The nuclei of the immature cells appeared larger and more centrally located as compared to mature cells, which have smaller nuclei with a more basal localization [31]. 

### 3.2. Transepithelial Transport and Uptake of ^109^Cd

**Figure 1:**
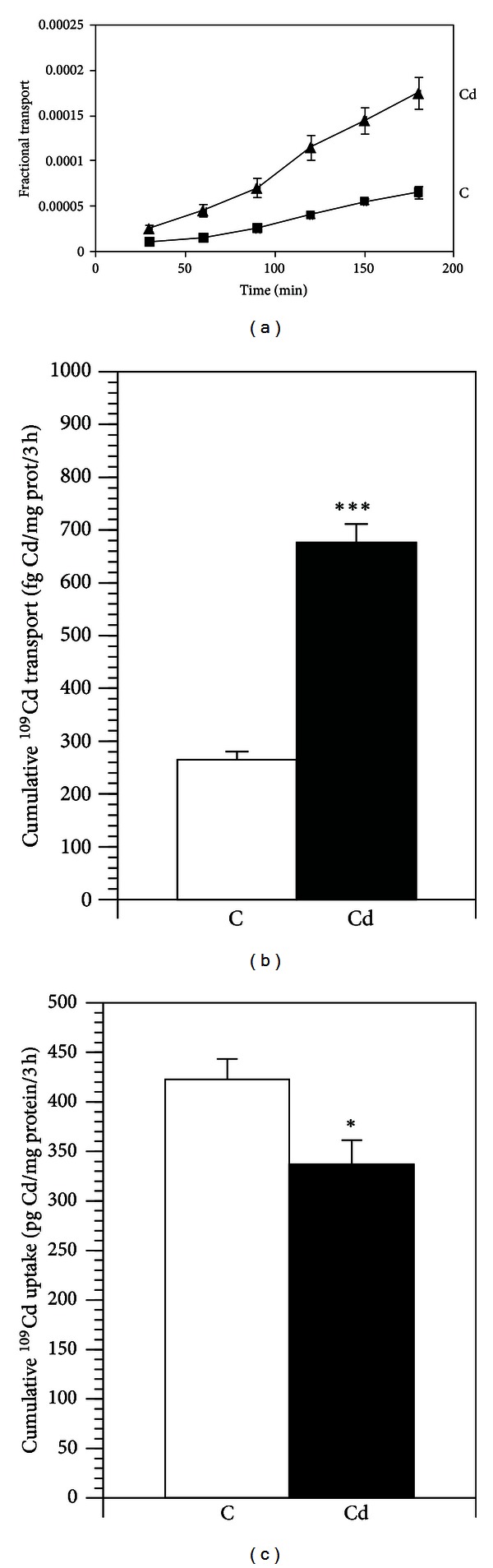
Fraction of ^109^Cd transported across immature Caco-2 cells from the apical to the basolateral chamber (a). Cumulative transport of ^109^Cd across immature Caco-2 cells (b). Apical uptake of ^109^Cd across into immature Caco-2 cells (c). Data are presented as mean ± SEM; *n* = 6; statistical significant difference compared to control; *P ≤ 0.05, ***P ≤ 0.001. Legends to the pretreatments: C: control; Cd: cadmium.

The transport of cadmium from the apical to the basolateral chamber across the Caco-2 monolayers was linear after an initial one-hour lag phase (Figure 1(a)). At each 30 min time interval, the transport of cadmium was higher across the cadmium pretreated monolayers as compared to the controls (Figure 1(a)). The cumulative cadmium transport from the apical to the basolateral chamber was increased by 170% in cells pretreated with cadmium (Figure 1(b)). Intracellular cadmium levels in cadmium pretreated cells was reduced by approximately 20% as compared to the controls at the end of the 3 h incubation period (Figure 1(c)).

### 3.3. Gene Expressions

**Figure 2:**
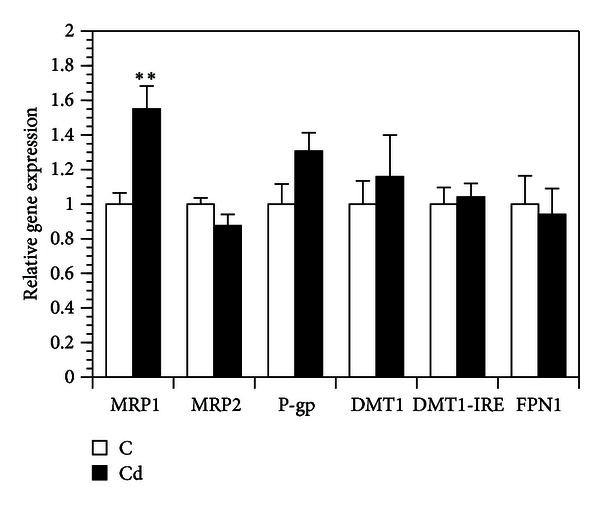
Relative gene expressions of MRP1, MRP2, P-gp, DMT1, DMT1-IRE, and FPN1 in immature Caco-2 cells. Data are expressed relative to controls, set at 1; mean ± SEM; *n* = 3; statistical significant difference compared to control; *P ≤ 0.05, **P ≤ 0.01. Legends to the pretreatments: C: control; Cd: cadmium.

The MRP1 gene expression in cells pretreated with cadmium was increased by 55% (Figure 2), whereas the MRP2 and P-gp mRNA levels were not significantly affected by cadmium pretreatment. The gene expressions of the iron transporters DMT1, DMT1-IRE, and FPN1 were not affected in the Caco-2 cells pretreated with cadmium (Figure 2). Gene expression of MT1 was increased 30 times after cadmium pretreatment (control: 1.0 ± 0.1; cadmium: 31.5 ± 2.1; mean ± SEM; *n* = 3; *P* ≤ 0.001). 

### 3.4. Functional Activity: MRP-Mediated Efflux of Calcein

**Figure 3:**
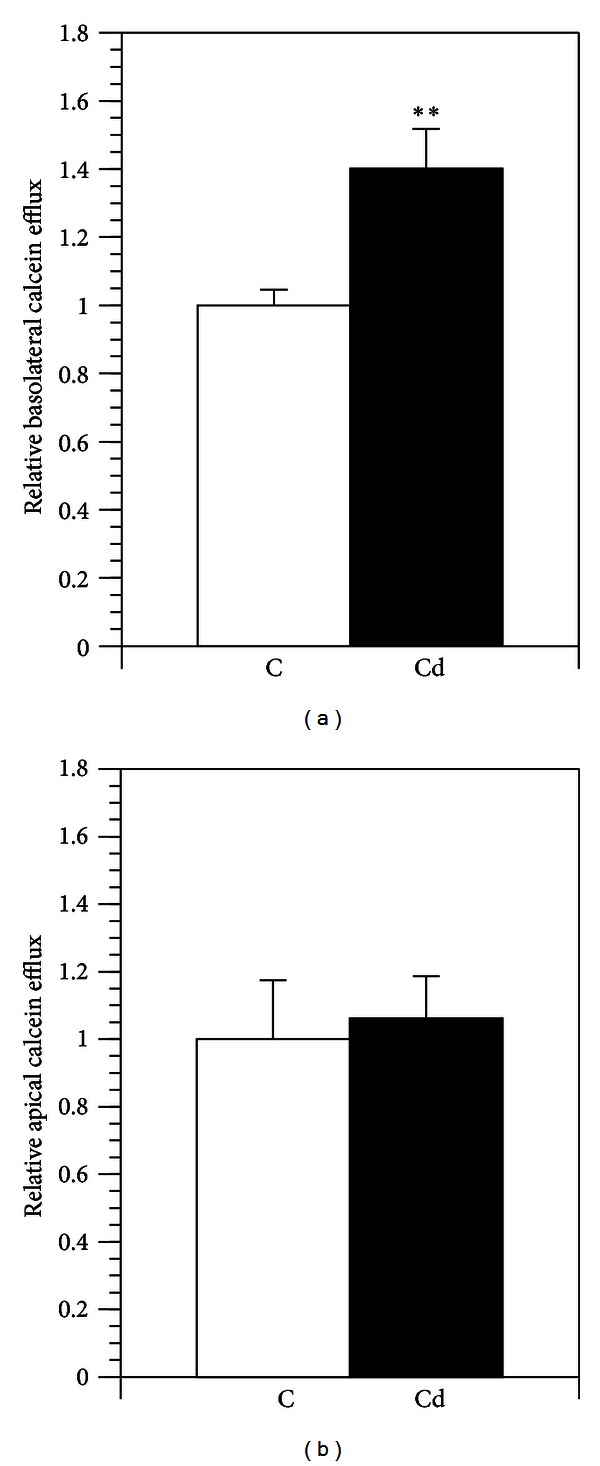
Relative basolateral (a) and apical (b) efflux of fluorescent calcein in immature Caco-2 cells. The MRP1 activity was examined by determining the cumulative level of fluorescent calcein effluxed into the basolateral chamber following two hours of incubation. Data are expressed relative to controls, set at 1; mean ± SD; *n* = 6; statistical significant difference compared to control; **P ≤ 0.01. Legends to the pretreatments: C: control; Cd: cadmium.

The basolateral efflux of calcein fluorescent dye was measured during two hours after one hour initial loading of the nonfluorescent dye calcein AM. Cadmium pretreatment significantly increased the efflux of calcein into the basolateral chamber (Figure 3(a)). The efflux of calcein into apical chamber was approximately four times higher than the efflux into the basolateral chamber. However, the pretreatment with cadmium did not affect the calcein efflux into the apical chamber (Figure 3(b)).

## 4. Discussion

The role of transporters in the high gastrointestinal uptake of cadmium in newborns is not fully understood [2, 17]. In addition, the cellular mechanism behind the increased intestinal absorption of cadmium following continuous exposure than after a single dose of the metal remains to be elucidated [25]. In the present study, we have demonstrated an upregulation of the basolateral efflux protein MRP1 in immature Caco-2 cells after cadmium pretreatment, both at the gene and at the functional level. The increased MRP1 gene expression and activity was correlated to an increased cadmium transport across the basolateral membrane, indicating that MRP1 is involved in this process. The augmented transport of the metal was not due to disrupted tight junctions as neither the passive diffusion of the paracellular marker mannitol nor the TEER was affected by cadmium. Cadmium is known to bind with high affinity to the thiol (SH) groups in GSH [28, 46], and it has been demonstrated in yeast that cadmium-GSH complexes can be transported by the MRP1 analogue YCF1 [21, 22]. Cadmium-GSH has also been shown to be transported by MRP1 in lung epithelial fibroblasts [47]. We suggest that cadmium, bound to GSH, is transported by MRP1 across the basolateral membrane in the immature intestinal cells, a transport that is upregulated by cadmium pretreatment. 

Our results showed a 30-fold increase in intracellular level of MT mRNA after cadmium pretreatment of the immature Caco-2 cells. Previously, a 5-fold increase in MT has been reported after long-term cadmium treatment of mature Caco-2 cells [26], indicating that immature cells are more sensitive to cadmium treatment and therefore induces MT expression more strongly. In concordance, Cardin et al. [35], demonstrated a higher induction of MT in immature Caco-2 cells compared to mature ones. These authors also reported that cadmium treatment decreased the thiol content by 65% in Caco-2 cells differentiated for 7 days, while no effects were observed on thiol content in mature Caco-2 cells, indicating developmental differences in the cell defense against cadmium-induced toxicity [35]. The reduced thiol content is in line with our suggestion of cadmium-induced formation of cadmium-GSH complexes, which are transported out of the cells by MRP1. 

Several studies have demonstrated that the iron transporters DMT1 and FPN1, upregulated at iron deficiency and repressed at iron sufficient conditions, are involved in cadmium transport in adult mammals [15, 16]. It has been suggested that there are ontogenic changes in the intestinal absorption and handling of iron in neonates and that iron absorption and expressions of DMT1 and FPN1 are not regulated until the time of weaning [18, 48]. Both iron transporters are exclusively located intracellularly at mid infancy and not until weaning present at the membranes of the duodenal enterocytes in rats [49, 50] and in pigs [17]. In suckling piglets, neither iron nor cadmium pretreatments have resulted in any effect on expression or localization of duodenal DMT1 or FPN1 [17]. In the present study, cadmium pretreatment did not affect the gene expression of DMT1 or FPN1 in the Caco-2 cells. Thus, the increased apical to basolateral cadmium transport in cadmium pretreated cells cannot be explained by an increased expression of these iron transporters.

Our results demonstrate that reduced apical uptake of cadmium into cadmium pretreated Caco-2 cells does not correlate to a reduced DMT1 or DMT1-IRE gene expression. This indicates that cadmium may be taken up by some other, non-DMT1-mediated, mechanism across the apical membrane of immature enterocytes and that cadmium pretreatment negatively affects this process. The reduced cadmium uptake after cadmium pretreatment may also in part be explained by the slightly increased gene expression of the apical efflux protein P-gp. It has been reported that long-term cadmium exposure increases P-gp expression and activity in mature Caco-2 cells [23]. In the present study, cadmium pretreatment did not affect gene expression or function of MRP2, which otherwise could have explained the reduced intracellular cadmium levels following exposure to the element on the apical side. The reduced cadmium uptake in the cadmium-treated immature cells did not correlate to the transport of the element into the basolateral chamber. However, it should be pointed out that the amount of cadmium transported across the basolateral membrane of the cells only constitutes a minor fraction of the total cellular uptake. Thus, even if the intracellular cadmium levels are lower in cadmium pretreated Caco-2 monolayers, the amount of metal, for example, MRP1 is still sufficient to saturate the transporter for extracellular efflux out of the cell into the basolateral compartment. 

Despite the strong MT induction, *in vivo* studies have demonstrated that MT only plays minimal roles in the gastrointestinal absorption of cadmium, though the retention of the metal in other tissues is MT dependent [27]. Accordingly, uptake of cadmium in the immature Caco-2 cells pretreated with cadmium was significantly reduced, indicating that MT is not involved in the retention of cadmium in neonatal enterocytes. 

In summary, our results showed that cadmium pretreatment of immature Caco-2 cells upregulated MRP1 both at the gene expression and functional levels, which correlated to an increased transport of the metal across the basolateral membrane. In addition, the two iron transporters DMT1 and FPN1 were not affected by cadmium and appeared not to be involved in the intestinal transport of cadmium in immature Caco-2 cells. In conclusion, our data indicate that continuous cadmium exposure increases the absorption of the metal in immature intestinal cells and that MRP1 conceivably is involved in the intestinal cadmium absorption in neonates. 

## Figures and Tables

**Table 1:** Primer sequences used for quantitative real-time PCR.

Primer	Oligo sequence	NCBI GenBank Accession No.	Product size (bp)
MRP1 forward	5′-GCAAATCCAGGAGACAGCTC-3′	NM_004996	113
MRP1 reverse	5′-TGATGTGCCTGAGAACGAAG-3′		
MRP2 forward	5′-CTGGTTGGGAACCTGACTGT-3′	NM_000392	172
MRP2 reverse	5′-CAACAGCCACAATGTTGGTC-3′		
P-gp forward	5′-GCTGTTAAGGAAGCCAATGC-3′	NM_000927	120
P-gp reverse	5′-AGCAATGGCGATTCTCTGTT-3′		
DMT1 forward	5′-CGTGGCGGATTGCAGGAGGA-3′	NM_001174126	124
DMT1 reverse	5′-ACGCTGACCACAGCAGCCAC-3′		
DMT1-IRE forward	5′-GCCATCAGAGCCAGTGTGTTTCT-3′	NM_001174125	198
DMT1-IRE reverse	5′-TGTCAGCTTTTCAAAGATCCCACC-3′		
FPN1 forward	5′-CGAGATGGATGGGTCTCCTA-3′	NM_014585	219
FPN1 reverse	5′-GGCTACGTCGAAAATGTGGT-3′		
MT1 forward	5′-GCAAATGCAAAGAGTGCAAA-3′	NM_005946	213
MT1 reverse	5′-ATGGGTCAGGGTTGTATGGA-3′		
